# Communication and school readiness abilities of children with hearing impairment in South Africa: A retrospective review of early intervention preschool records

**DOI:** 10.4102/sajcd.v66i1.604

**Published:** 2019-02-28

**Authors:** Ntsako P. Maluleke, Katijah Khoza-Shangase, Amisha Kanji

**Affiliations:** 1 Department of Speech Pathology and Audiology, School of Health Care Sciences, Sefako Makgatho Health Sciences University, South Africa; 2 Department of Speech Pathology and Audiology, School of Human and Community Development, University of the Witwatersrand, South Africa

## Abstract

**Background:**

The national prevalence of hearing impairment in South Africa is estimated to be four to six in every 1000 live births in the public health care sector. An undetected hearing impairment in childhood can lead to delayed speech and language development as well as put the child at risk of not achieving the necessary school readiness abilities that will enable them to achieve academic success. However, through early hearing detection and intervention services, children with hearing impairment can develop communication and school readiness abilities on par with children with normal hearing.

**Objective:**

The aim of the study was to describe communication and school readiness abilities of children who were identified with hearing impairment and enrolled in early intervention (EI) preschools in Gauteng.

**Methods:**

Within a descriptive research study design, a retrospective record review was conducted on files of eight children, ranging in age from 9 years and 7 months to 12 years and 7 months, identified with a hearing impairment and enrolled in EI preschools in Gauteng, South Africa. Descriptive statistics were used to analyse the data, using frequency distribution and measures of central tendency.

**Results:**

Current findings revealed that children with hearing impairment who were enrolled in EI preschools in Gauteng were identified late. This consequently led to delayed ages at initiation of EI services when compared to international benchmarks and the Health Professions Council of South Africa’s (HPCSA) guidelines of 2018. Consequently, participants presented with below average communication and school readiness abilities, which are characteristic of hearing impairment that is identified late.

**Conclusions:**

Transference of current contextually relevant research findings into practice by both the Department of Health and the Department of Basic Education forms part of future directions from this study. This conversion of research findings into service delivery must be conducted in a systematic manner at all levels in these two sectors to facilitate achievement of Early Hearing Detection and Intervention (EHDI), resulting in better communication and school readiness outcomes.

## Introduction

In South Africa, it is estimated nationally that the prevalence of hearing impairment is four to six in every 1000 live births in the public health care sector (Swanepoel, Storbeck & Friedland, 2009). More recently, studies specifically related to prevalence of hearing impairment in the Cape Town Metropolitan area and urban schools in Pretoria found that 11.4% of 174 children aged 0–3 years and 4.3% of 430 children aged 4–9 years presented with hearing impairment (Ramma & Sebothoma, 2016). Other findings from an urban South African context indicated that the youngest age group (<7 years of age) had a higher prevalence of hearing impairment (6.2%) when compared to the older age groups, which presented with a prevalence of 1.7% and 1.3% (Mohamed-Asmail, Swanepoel, & Eikelboom, 2016). These findings highlight the importance of the identification of hearing impairment as early as possible.

Evidence exists that has established a relationship between an undetected hearing impairment in childhood and delayed speech and language development, as well as social and emotional problems (Sininger, Grimes, & Christensen, 2010; van Dyk, Swanepoel, & Hall, 2015). These difficulties may result in lower educational and employment levels during adulthood (Joint Committee on Infant Hearing [JCIH], 2007). By its very nature, hearing impairment provides incomplete access to spoken language, leading to negative effects on the understanding and development of use of spoken language (Wang & Engler, 2011). Researchers have reported that as these children are at risk of not achieving the necessary skills to prepare them for school, this will have a negative impact on education and academic success, which will ultimately impact their ability to find employment later in their lives (Harrington, DesJardin, & Shea, 2009; Marschark, 2007). These difficulties may be further exacerbated by the realities of the South African context. A report from a 2013 meeting between the Department of Basic Education (DBE), Department of Public Service and Administration (DPSA) and the Deputy Minister of Women, Children and Persons with Disability indicated that the majority of deaf children did not have early access to early childhood development programmes for stimulation and development of sign language; the majority of deaf children were born to hearing parents, and without the necessary sign language development. In addition, approximately 80% of teachers in schools for the deaf were unable to use sign language. Few deaf learners were reported to have progressed beyond grade 12, with only a few schools having audiology equipment and audiologists (Parliamentary Monitoring Group, 2013).

Research has produced evidence that indicates that the speech and language abilities of children with hearing impairment can equal that of their hearing peers if the hearing impairment is identified early and intervention commenced timeously (Meyer, Swanepoel, & Le Roux, 2014; Swanepoel et al., 2009). According to the Health Professions Council of South Africa (Health Professions Council of South Africa [HPCSA], 2018) and the JCIH (2007), diagnostic audiological assessment should be conducted by 3–4 months of age with the commencement of early intervention (EI) services before 6 months of age. Through timeous initiation of EI services, children with hearing impairment can develop age-appropriate communication and school readiness abilities (DesJardin et al., 2014; Harrington, DesJardin, & Shea, 2010). Early intervention is used broadly to refer to intervention practices with children from birth to 5 years of age (Khoza-Shangase, Barrat, & Jonosky, 2010) who are identified as having, or being at risk of developing, a developmental delay (Ackah & Appiah, 2011). Early intervention is grounded in the conviction that during the first 3–5 years of life there is a unique opportunity to prevent or reverse children’s developmental delays (Fulcher, Nivelles, Purcell, Baker, & Munro, 2015).

Success in communication and school readiness abilities provides the foundation for children with hearing impairment to have similar educational opportunities to their normal hearing peers (Harrington et al., 2010; Le Roux, Swanepoel, Louw, Vinck, & Tshifularo, 2015). School readiness abilities equip a child to participate successfully on entering school (De Jager, 2014; Du Plooy, 2003) and enable them to learn easily, effectively and without emotional disturbance (Du Plooy, 2003). Although there is no clear statement of what factors constitute school readiness, most researchers in early childhood education agree that in addition to early communication abilities, a solid foundation in early literacy, attention skills and mathematical concepts supports later academic achievement in children (Harrington et al., 2009; Mukari, Ling, & Ghani, 2007).

Over the last decade, there has been a growing recognition of human rights for children with disabilities (Storbeck & Moodley, 2011). Access to education is one of a range of social citizenship rights that are intended to afford these children an opportunity to share in a basic level of social, economic, and cultural well-being and to mitigate inequalities (Storbeck & Moodley, 2011). Thus, there is a need for laying a solid foundation in early childhood to ensure adequate development and growth for children with hearing impairment (Storbeck & Moodley, 2011). The Children’s Act No. 38 of 2005 (as enacted in April 2010) makes provision for the delivery of early childhood development services that promote the development of children from birth to school-going age, and for programmes that provide developmentally appropriate learning and support (The United Nations International Children’s Emergency Fund [UNICEF], 2015).

Through EI services children with hearing impairment can be well prepared as they enter formal education (Harrington et al., 2009; Kanji & Khoza-Shangase, 2016). Thus, the current study aimed to describe the communication and school readiness abilities of children with hearing impairment who were enrolled in EI preschools in the Gauteng province, South Africa.

## Methodology

The research study set out to describe the communication and school readiness abilities of children with hearing impairment who were enrolled in EI preschools in Gauteng, South Africa. The following objectives were formulated:

- to determine the ages at identification of the hearing impairment and ages at initiation of EI services for children with a hearing impairment who were enrolled in EI preschools;
- to describe the communication abilities of children with a hearing impairment who were enrolled in EI preschools;
- to describe the school readiness abilities of children with a hearing impairment who were enrolled in EI preschools.

### Research design

A descriptive, retrospective record review design was adopted in the current study. A review of written and printed material of preschool files was undertaken to gather the relevant information for analysis, as outlined in the ‘Materials’ section.

### Participants

The primary population included eight hearing impaired children who had graduated from EI preschools. This sample had four males and four females with an age range of 9.7 years–12.7 years (median = 11.1 years) at the time of data collection. Upon enrolment at the EI preschools, participants’ ages ranged from 2 years to 5.8 years with a median of 2.4 years.

Six of the participants were white people and two were people of mixed race (Table 1). Four of the participants had been enrolled at EI Centre A, and the other four had been enrolled at EI Centre B. Early intervention Centre A is situated in Johannesburg and offers a specialised preschool programme for children with hearing impairments and/or language delay, while EI Centre B is situated in Pretoria and offers habilitation services for children with hearing impairments in a preschool setting (Maluleke, Khoza-Shangase, & Kanji, 2018).

**TABLE 1:** Demographic information of participants.

Participant number	Age (years, months)	Gender	Race
1	9.9	Female	Person of mixed race
2	10.8	Male	White person
3	11.7	Male	White person
4	10.9	Female	White person
5	9.7	Male	White person
6	12.7	Female	White person
7	10.3	Female	White person
8	12.5	Male	Person of mixed race

Participants had to meet the following criteria in order to be included in the study:

- Participants had to present with a diagnosis of a bilateral, moderate hearing impairment or greater, unaided.
- Participants had to be fitted with amplification devices, such as hearing aids and/or cochlear implants, in order to compensate for the hearing impairment.

Participants who presented with co-morbidities such as a cognitive impairment in addition to the hearing impairment were excluded from the study. Participants came from the upper middle-class to upper class socio-economic backgrounds.

### Procedure

In order to identify potential participants for the current study, the researchers obtained written consent from two EI preschools, allowing them access to the preschool files. Subsequently, the researchers compiled a list of caregivers’ contact details for all possible participants using purposive, non-probability sampling.

Primary caregivers of all possible participants were contacted by telephone, and permission was requested to review their child’s preschool records. Consent forms were then emailed to primary caregivers after they indicated their willingness to allow the researchers to review their child’s preschool records.

### Material

Data obtained from the preschool records included participants’ demographic information, audiology reports, age at commencement of EI services, communication abilities and school readiness abilities.

The communication abilities recorded for the purpose of this research study were the last available speech-language assessment results recorded prior to the participants’ graduation from the EI preschool. Participants’ communication abilities were assessed in English using standardised language assessment tools. School readiness abilities were extracted from the participants’ preschool reports issued upon graduation from the EI Centre.

To determine the ages at initiation of EI services, the researchers obtained results pertaining to both age at provision of amplification devices and age at provision of habilitation services.

### Data collection

The researchers (dually qualified speech-language therapists and audiologists) were not permitted to remove the files from the EI preschools, thus the desired data were recorded at the preschools on an Excel (2009) spreadsheet in such a way that the participants could not be identified by using participant reference numbers.

### Reliability and validity

The researchers ensured reliability by conducting the main study in the same manner as the pilot study which was used to ensure that the findings of the current study were reliable (Van der Riet & Durrheim, 2006).

Validity was ensured through alterations that were made to the data collection tool on the basis of the results of the pilot study.

### Data analysis

Data analysis followed descriptive statistics where measures of dispersion and central tendency were utilised, as the study was descriptive in nature (Creswell, 2009). These included the mean, mode and range.

## Ethical consideration

This study adhered to the ethical principles as outlined in the revised Helsinki Declaration of 1975 (World Medical Association, 2008), with ethical clearance obtained from the university’s Medical Human Research Ethics Committee (Protocol number: M130240).

## Results and discussion

### Ages at identification of the hearing impairment and initiation of early intervention services

Because all participants were identified late, ages at initiation of EI services were also consequently delayed (Table 2) (Maluleke et al., 2018). None of the participants received newborn hearing screening services, thus participants in the current study were identified late following maternal suspicion of the hearing impairment. Ages at identification of the hearing impairment ranged from 7 months to 4 years and 1 month, with a mean age at identification of 2 years and 3 months, and the median age was 2 years (Maluleke et al., 2018). Current findings are inconsistent with the recommendations by the HPCSA (2018) which suggests earlier targets for identification of hearing impairment.

**TABLE 2:** Ages at identification, amplification and initiation of early intervention services (*n* = 8).

Participant number	Age at identification (months)	Age at amplification (months)	Age at initiation of EI (months)
1	31	36	38
2	25	35	26
3	49	49	49
4	16	20	17
5	15	18	17
6	7	18	18
7	49	52	50
8	24	30	24

EI, early intervention.

The results of this study simulate those of other studies. Khoza-Shangase and Michal (2014) examined the current audiological management protocols for children with hearing impairment in the public hospitals of Gauteng, South Africa. In their results, Khoza-Shangase and Michal (2014) reported a mean age at identification of 1 year and 11 months, which is similar to findings in the current study. It is widely reported that children identified with a hearing impairment after 6 months of age may demonstrate a significant delay in milestones as compared to peers who are identified early on, which may result in delayed speech and language abilities throughout childhood (Abdala & Visser-Dumont, 2001). Hence, this finding pertaining to age at identification raises the necessity to develop effective and feasible Universal Newborn Hearing Screening (UNHS) throughout the diverse South African hospitals (Khoza-Shangase & Harbinson, 2015) and clinic settings (Petersen & Ramma, 2015) so that hearing impairment may be identified timeously (before parental suspicion), allowing for the commencement of EI soon after diagnosis.

The fact that delayed ages at identification of hearing impairment were found in the current study, it was to be expected that delayed age at provision of amplification devices would be found (Swanepoel, Johl, & Pienaar, 2013). Ages at provision of amplification devices ranged between 1 year and 6 months and 4 years and 4 months, with the median age at provision of amplification devices being 2 years and 8 months and the mode, 2 years and 6 months (Table 2). Reasons for delays in provision of amplification devices were not investigated as part of the current study and are implications for future research, but may be influenced by the limited number of audiologists in the public health care sector and long waiting lists as a result of constrained budgets.

Similar suboptimal provision of amplification devices in the South African context has been reported. In a national survey of paediatric audiology services conducted by Meyer et al. (2014), suboptimal provision of amplification devices at ages older than 2 years was reported. These findings demonstrate poor adherence to the HPCSA (2018) guidelines for best practice, which recommend provision of amplification devices within 1 month of identification of the hearing impairment. In addition to mandated UNHS, necessary budget provisions are required to ensure that children identified with a hearing impairment in South Africa are promptly provided with amplification devices. Evidence also suggests that provision of amplification devices as soon as possible after a child has been identified with the hearing impairment is of crucial importance as lack of auditory stimulation has an effect on the development of the child’s speech and language skills (Olusanya, Okolo, & Adeosum, 2004).

In addition to suboptimal provision of amplification devices, the current study found that commencement of EI services was late. Ages at initiation of EI services in the form of habilitation services ranged between 1 year and 5 months and 4 years and 2 months, with a mean age at initiation of services of 2 years and 5 months, and a median age of 2 years. These findings do not adhere to the HPCSA’s (2018) recommendation of infants with a confirmed hearing impairment receiving intervention services before 6 months and no later than 8 months of age. Research evidence shows that an infant with a hearing impairment who receives habilitation services within the first 6 months of life is likely to have linguistic, speech and cognitive development comparable to normal hearing peers, in contrast to persistent delays for those who are identified late. This benefit is attributed to the principle of ‘critical developmental period’, which allows for optimal cognitive, language and speech development early in the child’s life (HPCSA, 2007).

The findings of the current study, coupled with a lack of reports of studies conducted in the South African context investigating commencement of habilitation services for children with a hearing impairment, is of great concern. Owing to the financial constraints experienced by the South African health care system, implementation of Early Hearing Detection and Intervention (EHDI) services required adjustments from the developed context model in order to ensure that these programmes were contextually relevant and achievable (South African Speech-Learning-Hearing Association [SASLHA], 2007). Thus, studies in the South African context have mainly focused on validation of early hearing detection services and the development of contextually feasible models of service delivery (De Kock, Swanepoel, & Hall, 2016). Consequently, little information is available for studies that demonstrate the medium- to long-term benefits of the entire EHDI process from hearing screening to EI (Pillay, Moonsamy, & Khoza-Shangase, 2010).

### Communication abilities

**FIGURE 1:**
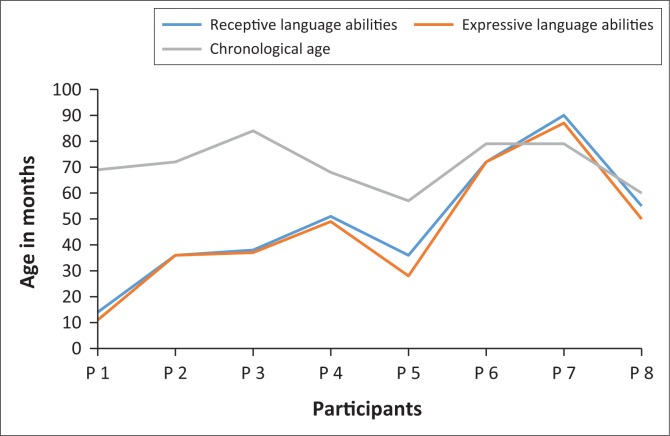
Participants’ communication abilities versus chronological age.

Communication abilities are reported in terms of receptive and expressive language compared to chronological age (Figure 1). One participant presented with above average communication abilities as measured by the Test of Auditory Comprehension of Language, 3rd edition (TACL-3) and Expressive One-Word Picture Vocabulary Test (EOWPVT). Two participants’ communication abilities approximated age-appropriate norms as measured by the Developmental Assessment Scale (DAS), while five participants presented with delayed receptive and expressive language abilities as measured by the DAS, TACL-3, EOWPVT and the Preschool Language Scale, 4th edition (PLS-4). Owing to the dearth of research in this area, there was a lack of results in the South African context to compare with the findings of the current study. However, the delayed communication abilities demonstrated by some of the participants were expected, on account of the late identification of the hearing impairment and subsequent late commencement of EI services reported in the current study (Maluleke et al., 2018).

In their study, Fulcher et al. (2015) investigated whether children with a hearing impairment who were identified early with no co-morbid conditions would outperform similar children who were identified late. Results of the study demonstrated that children identified early significantly outperformed the children who were identified late at all ages. Children identified before the age of 12 months achieved age-appropriate scores for speech as well as receptive and expressive language abilities. However, late identification and intervention, as demonstrated in the current study, misses the crucial 2 years of the language development period that will enable these children to develop age-appropriate language abilities (Storbeck & Young, 2016). This finding once again highlights the need for comprehensive EHDI infrastructure. Through such EHDI programmes, children with a hearing impairment can experience the optimal benefits from the earliest possible hearing identification and intervention (Meyer et al., 2014). It is possible for children with a hearing impairment to achieve age-appropriate speech and language outcomes (Fulcher, Purcell, Baker, & Munro, 2012).

### School readiness abilities

Participants’ school readiness abilities are reported based on attention/listening skills, communication abilities, mathematical concept knowledge and early literacy skills. A ‘yes’ indicates that the participant presented with age-appropriate skills for the area, while a ‘no’ indicates that the participant’s abilities for that area were not age-appropriate (Table 3).

**TABLE 3:** Participants’ school readiness abilities.

Expected abilities school readiness abilities	Question	P 1	P 2	P 3	P 4	P 5	P 6	P 7	P 8
Attention skills	-	No	No	No	Yes	No	Yes	Yes	No
	Concentrates on a task for at least 11 min?	-	-	-	-	-	-	-	-
Spoken language abilities	-	No	No	No	No	No	Yes	Yes	Yes
	Has a command of the language?	-	-	-	-	-	-	-	-
	Uses sentences to express ideas and needs?	-	-	-	-	-	-	-	-
Mathematical concepts	-	No	No	Yes	Yes	No	Yes	Yes	Yes
	Counts up to at least 10?	-	-	-	-	-	-	-	-
	Understands the concept of counting, sorting and grouping?	-	-	-	-	-	-	-	-
	Understands the concept of size, writes numbers?	-	-	-	-	-	-	-	-
	Counts up to at least 10?	-	-	-	-	-	-	-	-
Early literacy	-	No	No	Yes	No	No	Yes	Yes	Yes
	Names basic colours?	-	-	-	-	-	-	-	-
	Knows the letters of the alphabet?	-	-	-	-	-	-	-	-
	Knows the name and sounds of letters?	-	-	-	-	-	-	-	-
	Reads and writes the alphabet?	-	-	-	-	-	-	-	-

P, participant.

Only three participants attained age-appropriate school readiness abilities; however, the remaining five participants did not attain age-appropriate school readiness abilities as demonstrated by poor attention, communication abilities, concept knowledge and early literacy skills. The participants who had attained age-appropriate school readiness abilities also presented with communication abilities that were age-appropriate or approximated age-appropriate norms. This finding is consistent with Harrington et al.’s (2009) study, which reported that children who were identified with hearing impairment and received EI at a later date demonstrated lower language scores, which were related to lower school readiness scores.

This finding supports the belief that communication abilities are crucial in the development of age-appropriate school readiness abilities (Zaidman-Zait & Young, 2008). Presumably, children who have a solid foundation in communication abilities have the ability to apply their linguistic knowledge to concepts such as alphabet knowledge, colour recognition, number identification and time and sequence, which are all considered as skills necessary for school readiness (Harrington et al., 2009). The findings of the current study further illustrate that without EHDI programmes, infant and childhood hearing impairment are identified after critical language development periods have passed, resulting in limited opportunities to develop age-appropriate communication and school readiness abilities. Hence the need for comprehensive EHDI programmes which will meet the unique needs of children with hearing impairments and their families (Albino & Berry, 2013). According to Albino and Berry (2013), to forgo greater investment in ECD interventions means compromising the well-being of South Africa’s communities, perpetuating cycles of poverty, poor educational attainment, inequality and socio-economic challenges.

## Conclusion

Children with hearing impairment who were enrolled in EI preschools in Gauteng were identified late. This late identification consequently led to delayed ages at initiation of EI services. These delays were significantly beyond those stipulated in international benchmarks and HPCSA (2018) guidelines, where the ages preclude optimal development (Maluleke et al., 2018). Consequently, participants presented with below average communication and school readiness abilities, which are characteristic of hearing impairment that is identified late. These findings raise implications for systematic planning and implementation of international level gold standards at various levels of service delivery, in both the Department of Health and the Department of Basic Education. The consequences of this would be earlier identification of infant and childhood hearing impairment. Through early identification of hearing impairment and initiation of EI services, better communication and school readiness outcomes may be achieved. This will enable children with hearing impairment to perform on par with their normal hearing peers. Furthermore, urgent implementation of widespread EI services would serve as a concrete step to equalise opportunities for vocational and societal contexts for children with a hearing impairment.

### Strengths and limitations

This study may possibly be the first to look at communication in relation to school readiness abilities for EI preschool graduates in the South African context for children with hearing impairment. Available literature investigates these factors in isolation. Therefore, it addresses the dearth of research in this area.

The findings of the current study are reported and interpreted within identified limitations. A small sample size of eight participants from similar upper middle-class to upper class socio-economic backgrounds was used for the current study. Although not representative of the demographic profile of the country, it was representative of the context in which the study was conducted. Using a larger sample size with participants from varied backgrounds would have allowed for generalisability of the findings to the general population. Moreover, participants of the current study were all from the Gauteng province. Extending the research study to the rest of the eight provinces would have allowed the researchers to establish EHDI outcomes in South Africa as a whole, and hence raises an implication for future research.
